# The Cedar Project - Mobile Phone Use and Acceptability of Mobile Health Among Young Indigenous People Who Have Used Drugs in British Columbia, Canada: Mixed Methods Exploratory Study

**DOI:** 10.2196/16783

**Published:** 2020-07-27

**Authors:** Kate Jongbloed, Margo E Pearce, Vicky Thomas, Richa Sharma, Sherri Pooyak, Lou Demerais, Richard T Lester, Martin T Schechter, Patricia M Spittal, The Cedar Project Partnership

**Affiliations:** 1 School of Population and Public Health Faculty of Medicine University of British Columbia Vancouver, BC Canada; 2 The Cedar Project Prince George, BC Canada; 3 Wuikinuxv Nation Prince George, BC Canada; 4 Aboriginal HIV/AIDS Community-Based Research Collaborative Centre Victoria, BC Canada; 5 Cree Victoria, BC Canada; 6 Cree & Métis Surrey, BC Canada; 7 The Cedar Project Vancouver, BC Canada; 8 Division of Infectious Diseases Faculty of Medicine University of British Columbia Vancouver, BC Canada

**Keywords:** Indigenous, mobile health, mHealth, text messaging, substance use, HIV/AIDS

## Abstract

**Background:**

Indigenous leaders continue to be concerned about high rates of HIV and barriers to HIV treatment among young Indigenous people involved in substance use. Growing evidence suggests that using mobile phones for health (mHealth) may be a powerful way to support connection with health services, including HIV prevention and treatment.

**Objective:**

This study examined the patterns of mobile phone ownership and use among young Indigenous people who have used drugs living with or vulnerable to HIV and explored the acceptability of mHealth to support access to health care in this population.

**Methods:**

The Cedar Project is a cohort study involving young Indigenous people who have used drugs in Vancouver and Prince George, British Columbia. This mixed methods exploratory study involved 131 Cedar Project participants enrolled in our WelTel mHealth program. At enrollment, participants completed a questionnaire related to mobile phone use and interest in mHealth. Data were linked to Cedar Project questionnaires and serodata. We present comparative statistics (quantitative) and results of a rapid thematic analysis (qualitative) related to mobile phone patterns and interest in receiving mHealth.

**Results:**

Less than half of the participants (59/130; 45.4%) reported owning a phone. Among those with a phone, the majority owned a smartphone (46/59; 78%). Most participants with a phone reported having an unlimited texting plan (39/55; 71%), using the internet on their phone (44/59; 75%), and texting daily (44/55; 80%). A majority reported that using a mobile phone for health would be invaluable (120/130; 92.3%). There were no differences in mHealth acceptance between participants who owned a phone and those who did not (*P*>.99). All but one participant living with HIV felt using a mobile phone would be helpful for their health, while a small proportion of HIV-negative participants remained unsure (1.9% vs 11.7%; *P*=.047). In response to open-ended questions asking why using a mobile phone may be helpful for health, participants identified a diverse set of anticipated benefits: (1) connection for emotional, mental, and spiritual support, (2) connection to family, (3) staying in touch and/or being reachable, (4) overcoming current barriers to phone use, (5) convenience, privacy, and safety, and (6) access to health care and emergency services.

**Conclusions:**

We observed high acceptance and interest in using mobile phone technology for health despite low rates of personal mobile phone connectivity among young Indigenous people who have used drugs living with and vulnerable to HIV in British Columbia, Canada. Mobile phones were viewed as a way to support connections and relationships that are seen as critical to health and well-being among young Indigenous people in this study. Findings may be useful for health care providers preparing to scale up mHealth programs to support HIV prevention and treatment in this population.

## Introduction

Researchers have started to understand mobile phones as a necessity comparable to other utilities and argue that gaps in access may re-enforce or exacerbate other disparities, including those related to health [1,2]. However, marginalized groups may be excluded from access due to structural inequalities (including health, economic, and gender disparities) that create a “digital divide,” or differential access to digital technologies [3]. Mobile phones have been identified as a critical resource for individuals who require regular contact with health care providers, social services, and social support [2,4,5]. Conversely, interruptions in phone connectivity may disrupt important health-related communication networks, such as contact with health care providers, social services, and social support [2].

Health care providers have begun to embrace the potential of widespread mobile phone usage by offering mobile health (mHealth) programs: mobile phone-based interventions that aim to improve health outcomes among clients experiencing a variety of health conditions. These mHealth interventions utilize mobile phone functions such as calling, texting, and/or smartphone apps, and may be used to provide reminders, information, or support. mHealth initiatives utilizing text messaging have been found to be successful in supporting engagement in health care for people living with HIV [6-14]. Such initiatives can facilitate real-time problem solving between patients and health care providers when medication, health, or other issues arise; remind clients to take medications, attend appointments, and take care of their health; and demonstrate that “somebody cares” [7,15,16]. More recently, mHealth initiatives have aimed to address substance use as well as optimize and expand treatment for substance use and HIV [17-22]. Notably, there is a paucity of evidence of mobile phone use for health and mHealth interventions for Indigenous people living with or at risk of HIV, including those using drugs [10].

Indigenous scholars and leaders continue to be concerned about high rates of HIV and other harm among Indigenous young people who use drugs resulting from the ongoing impacts of colonization [23,24]. Substance use, poverty, barriers to health care access, and limited mobile phone ownership may be mutually reinforcing, leading to further marginalization from care and services. Despite the potential to connect young Indigenous people who have used drugs to health services, little is known about mobile phone access and acceptability of mHealth programs for this key population. This study (1) examines patterns of mobile phone ownership and use among young Indigenous people who have used illicit drugs in British Columbia and (2) explores the acceptability of mHealth for this population.

## Methods

### Study Design and Setting

This is a mixed methods cross-sectional analysis involving young Indigenous people who have used drugs participating in the Cedar Project WelTel mHealth Study. The study took place in inner-city settings of two cities in British Columbia, Canada. Vancouver is a large city in southern British Columbia on Coast Salish territory. In 2016, nearly 14,000 Indigenous people lived in Vancouver, accounting for 2.2% of the population [25]. Prince George is a forestry and mining town in British Columbia’s Northern Interior on the traditional territory of the Lheidli T’enneh people. Just over 11,000 Indigenous people lived in Prince George in 2016, accounting for 15.4% of the population [26]. Both cities are home to large “away from home” Indigenous populations with people from nations and territories across the province and country.

### Participants

The Cedar Project is a cohort involving 782 young Indigenous people who have used drugs in Vancouver and Prince George, British Columbia. The term Indigenous is used as Cedar Project participants represent many of the diverse First Nations, Inuit, and Métis communities across Canada and often live far away from their home communities. Methods have previously been described in detail [27]. Briefly, participants were recruited through health care providers, street outreach, and word of mouth. Initial recruitment took place between 2003 and 2007 and reopened in 2011. Participants were eligible if they self-identified as Indigenous, were between 14 and 30 years of age, and had smoked or injected illicit drugs (other than cannabis) in the month before enrollment. Drug use was confirmed using saliva screens (ORAL-screen, Avitar Onsite Diagnostics). Follow-up interviews were carried out every 6 months, and blood samples were collected for HIV and hepatitis C antibody tests.

In September 2014, the Cedar Project WelTel mHealth Study was initiated. The mHealth program consisted of a structured mobile phone initiative to connect young Indigenous people who have used drugs with Cedar Case Managers in a community-based setting. It included a package of supports, including a mobile phone and cellular plan, alongside weekly two-way text messaging and support from Cedar Case Managers. Each Monday at noon, a text message saying, “how’s it going?” was automatically sent to participants through the WelTel mHealth platform. Cedar Case Managers responded to all participants and followed up with participants who replied with a specific problem or need. On Wednesday, those who had not replied received an additional text saying, “Haven’t heard from you, are you ok?” On Thursday or Friday, Case Managers attempted to call all remaining participants who had not responded to the text message. The program was offered between September 2014 and January 2016. Of the 60 HIV-positive participants in the Cedar Project Blanket Case Management study, 52 (88.3%) agreed to participate in the mHealth study. In addition, with the aim of recruiting 94 HIV-negative Cedar Project participants, 131 were randomly selected to be invited, of whom 79 (78.7%) agreed to join. Thus, 131 Cedar Project participants were enrolled in the mHealth study and provided a mobile phone and plan, weekly text messaging, and connection to a Case Manager. They also continued with their regular visits to the main Cedar Project cohort study.

### Data Sources

At enrollment into the mHealth study, participants completed a short questionnaire on mobile phone use, which is the focus of the analysis presented here. Close- and open-ended questions were related to interest in and concerns about using mobile phones and text messaging for health. Data were linked with questionnaires and serodata collected every 6 months as part of the main Cedar cohort. Time-independent variables were obtained from baseline questionnaires and time-dependent variables were collected from the follow-up visit that occurred closest to (but <30 days after) the mHealth baseline visit.

### Analytical Approach

We conducted descriptive analyses related to phone ownership and patterns, and participants’ interest and concerns using mobile phones for health. Differences in characteristics and acceptability of mHealth by phone ownership and HIV status were compared using the Chi-square and Fisher exact tests (dichotomous variables), and *t* tests and Mann-Whitney-Wilcoxon Test (continuous variables). All *P* values are 2-sided. Analyses were performed using R version 3.5.0 (R Foundation for Statistical Computing) [28].

Short responses (1-40 words) to open-ended questions were recorded verbatim. Using a rapid qualitative analysis approach [29], two authors independently read and reread responses to identify recurring themes. Emerging themes were discussed, defined, and a coding manual was created. Responses were sorted into categories using the NVivo software Version 10 [30]. Representative responses were chosen to highlight the themes.

### Ethical Considerations

The Cedar Project follows the guidelines provided in the Tri-Council Policy Statement on Ethical Conduct for Research Involving Humans—Chapter Nine: Research involving the First Nations Inuit and Métis Peoples of Canada [31] and adheres to the principles of Ownership, Control, Access, and Possession [32]. The Cedar Project Partnership, an independent body of Indigenous Elders, leaders, and health experts, governed all aspects of this study. The study was approved by the University of British Columbia/Providence Health Care Research Ethics Board. All participants gave both verbal and written consent, and it was emphasized that deciding not to participate in this substudy would not affect continued involvement with Cedar or support from staff.

## Results

### Baseline Characteristics

More than half (81/131, 61.8%) of mHealth participants were women, and half of mHealth participants lived in Prince George (65/131, 49.6%; Table 1). Approximately half (63/130, 48.6%) had a parent who attended residential school, and substantial proportions had been apprehended from their parents (100/131, 76.3%) and/or experienced childhood sexual abuse (70/123, 56.9%). Among parents, 53.5% (61/114) reported that they had ever had a child apprehended. Fewer participants reported connection to Indigenous cultures either in the past or present, including having a traditional language spoken at home (54/130, 41.5%), speaking a traditional language (53/130, 40.8%), often/always speaking a traditional language today (4/130, 3.1%); participating in ceremony (30/130, 23.1%); recently accessing traditional food (63/130, 48.5%); and living by traditional culture (22/127, 17.3%). Recent involvement in sex work was reported by 16.9% (15/89) participants, and recent injection drug use was reported by 44.2% (57/129) participants. Overall, 60.3% (79/131) and 40.5% (53/131) participants were living with hepatitis C and HIV, respectively.

**Table 1:** Characteristics of young Indigenous people who have used drugs enrolled in the Cedar Project WelTel mHealth Program by phone ownership.

Characteristic		Total (n=131)		No phone (n=71)			Own a phone (n=59)			*P*^a^ value	
		N	n (%)		N	n (%)		N	n (%)		
**Demographics**											
	Age (years), median (IQR)	131	33 (30-36)		71	34 (31-36)		59	33 (29-36)		.26
	Sex (female)	131	81 (61.8)		71	41 (57.7)		59	39 (66.1)		.43
	Location (Prince George)	131	65 (49.6)		71	29 (40.8)		59	35 (59.3)		.05
	In a relationship	129	33 (25.6)		70	19 (27.1)		58	14 (24.1)		.85
	Sexual identity (LGBTQ^b^)	131	23 (17.6)		71	7 (9.9)		59	16 (27.1)		*.02*
	Education (Did not graduate high school)	129	106 (82.2)		69	58 (84.1)		49	47 (79.7)		.68
	Recent^c^ homelessness	131	34 (26.0)		71	17 (23.9)		59	17 (28.8)		.67
	Recent housing instability	126	56 (44.4)		69	32 (46.4)		56	24 (42.9)		.83
	Recent incarceration	128	19 (14.8)		69	13 (18.8)		58	6 (10.3)		.28
**Cultural Connection & Resilience**											
	Traditional language spoken often at home growing up	130	54 (41.5)		71	34 (47.9)		58	19 (32.8)		.12
	Speak traditional language (*yes or a bit*)	130	53 (40.8)		71	32 (45.1)		58	20 (34.5)		.30
	Often/always speak traditional language today	130	4 (3.1)		71	2 (2.8)		58	1 (1.7)		>.99
	Ever participated in traditional ceremonies	130	30 (23.1)		71	12 (16.9)		58	17 (29.3)		.14
	Often or always live by traditional culture	127	22 (17.3)		70	12 (17.1)		56	9 (16.1)		>.99
	Recent access to traditional food	130	63 (48.5)		71	30 (42.3)		58	32 (55.2)		.20
	Resilience, mean (SD)	122	63.37 (21.35)		65	61.37 (20.05)		57	65.19 (22.75)		.33
**Trauma**											
	Either parent at residential school	130	63 (48.5)		71	39 (54.9)		58	24 (41.4)		.18
	Apprehended from biological parents	131	100 (76.3)		71	54 (76.1)		59	46 (78.0)		.96
	Childhood sexual abuse (≤13)	123	70 (56.9)		66	30 (45.5)		56	39 (69.6)		*.01*
	Ever attempted suicide	129	44 (34.1)		69	22 (31.9)		59	21 (35.6)		.80
	Ever had a child apprehended^d^	114	61 (53.5)		61	28 (45.9)		52	33 (63.5)		.09
**Sexual vulnerability**											
	Recent sex work^e^	15/89	16.9%		44	8 (18.2)		44	7 (15.9)		>.99
	Recent sexual assault	5/129	3.9%		70	2 (2.9)		58	3 (5.2)		.66
**Substance use**											
	Recent injection drug use	129	57 (44.2)		69	34 (49.3)		59	23 (39.0)		.32
	Ever overdosed	130	52 (40.0)		71	29 (40.8)		58	23 (39.7)		>.99
	Recent alcohol/drug treatment	130	51 (39.2)		71	29 (40.8)		58	22 (37.9)		.88
	Current methadone treatment^f^	66	38 (57.6)		38	25 (65.8)		28	13 (46.4)		.19
	Ever tried to quit drugs/alcohol	129	109 (84.5)		70	55 (78.6)		58	53 (91.4)		.08
**Health outcomes**											
	HIV infection	131	53 (40.4)		71	35 (49.3)		59	18 (30.5)		*.046*
	Hepatitis C virus infection	131	79 (60.3)		71	46 (64.8)		59	33 (55.9)		.40
	Psychological distress, mean (SD)	131	1.00 (0.84)		71	1.04 (0.90)		59	0.98 (0.77)		.71
	Recent hospitalization	131	15 (11.5)		71	5 (7.0)		59	10 (16.9)		.10

^a^*P* values indicated in italics are statistically significant.

^b^LGBTQ refers to lesbian, gay, bisexual, transgender, queer or questioning sexual identities.

^c^Recent refers to the 6-month period prior to the interview.

^d^Among a subset of people who were parents (n=114).

^e^Among a subset of people who said yes to having sex in the past 6 months (n=91).

^f^Among a subset of people who said yes to ever being on methadone (n=66).

### Patterns of Mobile Phone Use

Slightly less than half (59/130, 45.4%) of the participants reported owning a phone at baseline. Among those, the majority owned a smartphone (46/59, 78%), had an unlimited texting plan (39/55, 71%), used the internet on their phone (44/59, 75%), and texted daily (44/55, 80%; Table 2). No differences in the patterns of mobile phone use were observed between men and women. Those who identified as lesbian, gay, bisexual, transgender, queer or questioning (LGBTQ), or reported experiencing childhood sexual abuse, were more likely at enrollment to own a phone, while people living with HIV were less likely to own one (Table 1).

**Table 2:** Baseline mobile phone use patterns among young Indigenous people who have used drugs who reported owning a phone at enrollment into the Cedar Project WelTel Mobile Health Study (N=59).

Mobile phone use pattern		Value, n (%)	
**Type of phone (n=59)**			
	Basic		13 (22)
	Smart		46 (78)
**Texting plan (n=59)**			
	Yes		53 (89)
	No		6 (10)
**Type of text plan (n=55)**			
	Pay as you text		12 (21)
	Unlimited		39 (71)
	Limited		2 (3)
	Unsure		2 (3)
**Access internet on phone (n=59)**			
	Yes		44 (75)
	No		15 (25)
**Frequency of texting (n=55)**			
	Never		3 (5)
	Rarely (1× per month)		2 (3)
	Occasionally (1× per week)		0 (0)
	Frequently (2-3× per week)		6 (10)
	Very frequently (daily)		44 (80)

### Mobile Health Acceptance

Participants were asked whether they felt using a mobile phone would be helpful for health care and if they had any concerns using text messaging for their health (Table 3). A majority reported that using a mobile phone for health would be invaluable (120/130, 92.3%). There were no differences in mHealth acceptance among participants who owned a phone and those who did not. All but one participant living with HIV felt using a mobile phone would be helpful for their health, while some HIV-negative participants remained unsure (1.9% vs 11.7%; *P*=.047). No differences in concerns using text messaging for health were observed between those living versus not living with HIV.

**Table 3:** Self-reported mHealth acceptance stratified by phone ownership and HIV status (N=130).

Mobile health acceptance		Total (n=130), n (%)	Phone ownership			HIV status		
			Yes (n=59), n (%)	No (n=71), n (%)	*P* value	HIV+ (n=53), n (%)	HIV- (n=77), n (%)	*P* value
**Do you think using a cell phone would help with your health care and be helpful to you?**								**.047**
	Yes	120 (92.3)	55 (93.2)	65 (91.5)	>.99	52 (98.1)	68 (88.3)	
	No	0 (0)	0 (0)	0 (0)		0 (0	0 (0)	
	Unsure	10 (7.7)	4 (6.8)	6 (8.5)		1 (1.9)	9 (11.7)	
**Do you have any concerns about using text messaging for your health care?**								**.45**
	Yes	4 (3.1)	2 (3.4)	2 (2.8)	>.99	1 (1.9)	3 (3.9)	
	No	125 (96.2)	57 (96.6)	68 (95.8)		51 (96.2)	74 (96.1)	
	Unsure	1 (0.7)	0 (0)	1 (1.4)		1 (1.9)	0 (0)	

### Benefits of Phone Use for Health

The survey included an open-ended question asking why using a mobile phone may be helpful for health. Nearly all participants (127/130, 97.7%) responded, suggesting a diverse set of anticipated benefits. Analysis of these responses revealed 6 themes: (1) connection for emotional, mental, and spiritual support, (2) connection to family, (3) staying in touch and/or being reachable, (4) overcoming current barriers to phone use, (5) convenience, privacy, and safety, and (6) access to health care and emergency services.

#### Connection for Support

Many participants (29/127, 22.8%) anticipated that a mobile phone would enable them to reach out or be contacted for emotional, mental, and spiritual support. Supporters included health professionals, “workers,” spouses, friends, and family. One participant explained,

I am a social person…I constantly stay in contact with people so I don't get depressed.

Female (F), Prince George (PG)

Another said,

Having someone to talk to or someone to call. That helps.

F, Vancouver (Van)

A third said,

Communicating, letting people know how you are, what mood you’re in…if they know you’re in a bad mood they might call you.

M, Van

Having support reducing or abstaining from substance use was how 4.7% (6/127) participants anticipated using or currently used their phone for support. Participants felt that they could use the phone to call their 12-step/recovery sponsor, remember to take their methadone, access detox, and/or avoid a relapse. For example, one participant explained:

My mental health hasn’t been all that good lately but having someone to talk/text with will be better than turning to the bottle or needle. I won’t be so alone.

F, Van

#### Connection to Family

Connection to family was identified as an important potential health benefit by 8.7% (11/127) participants. One described how having a phone would facilitate in-person visits with family:

So I can get ahold of family more and probably go see them for Christmas!

M, Van

A total of 5.5% (7/127) participants anticipated benefits related to pregnancy and parenting, including being able to stay in touch with social workers, receiving health care leading up to and during labor, and calling for help in case of emergency.

#### Staying in Touch

Participants also spoke about the importance of “staying in touch” more broadly, suggesting that the benefit of a mobile phone was connection in and of itself. Some spoke about how a phone would help reduce feelings of isolation and allow them to, “keep connected with the world.” One person described how,

If I don’t have a phone I feel cut off, I get anxiety.

F, Van

Another said,

It’s nice to have someone check up on you sometime.

F, PG

Eight (8/127, 6.3%) participants described the value of being more “reachable”, as being unreachable was perceived as a source of stress for themselves and people around them. As one participant put it,

I don’t have to stress—people can reach me. The phone is my lifeline.

F, PG

#### Overcoming Current Barriers to Phone Use

A number of participants spoke of barriers they currently faced with accessing a phone or making calls. Many who did not currently have a phone relied on borrowed phones, including those belonging to friends, in the lobby of their building, or available at local service agencies. Borrowing phones had drawbacks including time and energy it took to find a phone, time limits on phone calls, inability to receive messages or a call back, lack of privacy, and potential for stigma or disclosure. One participant explained the impact of time limits:

You have to get hold of people right away or it doesn’t work out.

F, Van

Another described concerns when calling from service agencies, especially those known to serve people who use drugs:

If you call from [Vancouver’s supervised injection facility], it has call display…then people know that you’re a drug user.

M, Van

#### Convenience, Privacy, and Safety

Some participants (16/127, 12.6%) indicated that having a mobile phone would make communication more convenient, including reducing the need to seek support on foot and save you “the mileage on your shoes” [M, PG]. For 2.4% (3/127) participants, privacy was an important anticipated benefit of having their own phone. One explained that owning her own phone would provide more control over the circumstances of engagement with health care providers, whereas currently her partner was too involved:

I can talk to my doctor one-on-one without having [partner] follow me or know everything.

F, PG

In addition, some participants felt that having a phone would afford some degree of safety, including from violence in relationships and street life.

#### Access to Health Care and Emergency Services

More than 20% of participants expected to use the study phones, or currently used their own mobile phones, to connect directly to health care, including calling doctors, nurses, and/or counselors, arranging out-of-town medical care, making appointments, organizing rides, and receiving messages from health services. A total of 11.0% (14/127) participants also planned to use their phones to receive medication and appointment reminders, including reminder calls from health care providers or setting a reminder on the phone itself. Owning a mobile phone was also seen as a potentially important resource in the case of physical or mental health emergencies faced by the participants themselves or those around them.

### Concerns About Using Texting for Health

Few participants (4/127, 3.1%) had concerns about using text messaging for health (Table 3). In total, 2.4% (3/127) participants spoke about anticipated challenges with mobile phone technology, including poor eyesight and low literacy, making it difficult to read and respond to text messages; 1.6% (2/127) participants reported fear that mobile phones may cause cancer. One participant raised issues about confidentiality, explaining that it may be hard to confirm,

that you’re talking to the right person.

M, Van

However, others spoke about how confidentiality was not a concern because either they did not feel they had serious health concerns or they would take steps to protect their privacy. For example, 1 participant said,

No. Nobody’s going to be looking at my phone. Even if they try there’ll be a password.

F, Van

## Discussion

### Patterns of Mobile Phone Ownership

To our knowledge, this is the first study to report on patterns of mobile phone ownership and use among young Indigenous people who have used drugs in Canada. Less than half (45.4%) of the participants in this study owned a mobile phone. Observed phone ownership was considerably lower than rates in Canada and North America in general, and similar or lower compared with other marginalized groups, including street-involved youth (45%-63%), homeless adults (44%-78%), and people who use drugs (83%-86%) [33-38]. Many young Indigenous people have been acutely affected by colonization, including having parents and family members who were forced into residential schools, removal from family into the child welfare system, and experiences of childhood sexual abuse [39,40]. Some have turned to substance use as a way to cope with the effects of these historical and lifetime traumas [40-42]. Intersections of substance use and poverty can create barriers to connectedness, for example, by contributing to incarceration and housing transitions [43]. Lack of phone ownership must be understood within the context of colonization, which continues to impact the well-being of Indigenous people across Canada and is a key consideration for future mHealth programs [44].

Despite low phone connectivity, a majority of participants, including those living with HIV, felt that using a mobile phone for their health would be helpful. Mobile phones were viewed as a way to support social connections, which may reflect an Indigenous worldview that highlights connectivity, relationships, interconnectedness, and interdependence as critical to health and well-being [45-50]. This was demonstrated in participants’ emphasis on the role of mobile phones as a way to re/connect with family, despite experiences of childhood trauma and, often, many years of separation [39-42]. Three-quarters (76%) of the participants had been taken from their parents into the child welfare system, and as a result, many lived far from their home communities. Yet, despite experiencing traumatic separations, family connections remain a powerful source of strength and resilience among young Indigenous people, as highlighted in other studies [51,52]. From a wholistic perspective of health and well-being, phones may provide an important way of connecting with family and loved ones, especially when living far from home.

The vital role of family in health and well-being has also been identified specifically among Indigenous people living with HIV [24]. Family relationships can be an important source of strength, a foundation of emotional support, and a motivator to stay healthy [53-55]. Family members, including partners, parents, and children often provide primary or first line support [55], helping to meet the logistical and emotional demands of dealing with diagnosis [56], initiating care [57], and adhering to medication [58]. Among Cedar participants living with HIV, access to mobile phones may enhance access to family networks who may provide social, material, and emotional support as they navigate complex health issues, systems, and treatment regimens [59].

Mobile phones were also seen as an opportunity to support health and well-being in the context of pregnancy and parenting. Previous Cedar research among young Indigenous mothers observed that being able to parent their children was key to participants’ own wellness, while those whose children had been taken into care expressed feelings of deep regret and loss [41]. Participants in this study included pregnant mothers preparing for birth, parents of children currently in care of the state who were interested in visitation and regaining custody, and parents with custody of children who were navigating ongoing relationships with social workers as well as trying to protect their children’s health and safety. Participants identified diverse ways in which being connected by phone would support carrying out their responsibilities as parents as well as nurturing their connections with children in care.

### Implications of Phonelessness for Health and Wellness

Indigenous people who use drugs have frequently encountered systemic and interpersonal racism, stigma, and judgment within harm reduction and health services [23,54,60-62]. As noted by other researchers, participants in this study reflected that periods of “phonelessness,” contribute to substantial burdens of time and energy required to remain in contact with services through in-person visits or borrowing a phone [2]. These efforts are not always successful and can result in missed follow-up calls, appointments, and test results [2]. Cedar participants anticipated that having a mobile phone would support access to health care, such as allowing them to speak directly with a provider (doctor, clinic, nurse, pharmacist, and counsellor), coordinate appointments, and visit logistics, or call 911 in an emergency. Participants also viewed mobile phones as potential tools for accessing emotional, mental, and spiritual support from a diverse group of care providers when experiencing crises related to unaddressed trauma, substance use, and/or mental health challenges. This may reflect a desire to access what Indigenous scholars have described as “relational care,” which emphasizes connections and takes a “whole person” perspective of well-being [54].

The impact of phonelessness on overall health may be especially pronounced in the context of HIV care. Findings from our recent systematic review highlighted profound gaps in access to the HIV cascade of care among Indigenous peoples [24]. Emerging evidence also indicates that barriers to phone access may be associated with poorer health outcomes among people with HIV [38]. However, our study indicates that young Indigenous people living with HIV are especially interested in receiving mHealth support to enhance engagement with health care. Others have shown that mHealth programs may improve relationships between health care providers and patients over time [16,63], and that strong patient-provider relationships that are engaging, validating, and emphasize partnership are more likely to facilitate engagement and retention in HIV care [64]. For young Indigenous people who use drugs, mHealth programs that take a culturally safe approach, including avoiding judgment of drug use and honoring Indigenous identities, may help to strengthen relationships with health care providers and engagement in care [65,66].

A mobile phone may also be vital for the safety of young Indigenous people who use drugs, in the face of housing instability [43], police surveillance [29], transitions into injection [67], sexual assault [68], suicide [69,70], and overdose [70]. A qualitative study of 43 street-involved youth in Seattle observed that mobile phones have become intrinsic to safety [71]. Among 100 homeless people in Philadelphia, participants described how with access to a mobile phone, “help is a phone call away,” especially in the context of threats to health and safety [35]. Cedar participants discussed how phones would support their safety by enhancing their privacy and enabling them to assist others by calling 911 in an emergency. Mobile phones may also be an important source of safety information for people who use drugs by allowing them to subscribe to relevant text message alerts [35]. For example, the Vancouver Community Network Street Messaging System sends emergency text alerts such as extreme weather shelter openings or “bad batch” incidents to enrolled residents [72]. Closely related to safety is the impact of not having a phone on privacy. This may be of particular concern for people living with HIV or using substances in the context of persistent stigma and criminalization. For example, Gonzales et al described how reliance on borrowed phones by 29 low-income clients of an HIV clinic created a risk of HIV disclosure, such as if the clinic returned a call or if the owner of the phone was present during the call [2]. For one of our participants who relied on her partner’s phone, having a phone of her own presented an opportunity to speak to her doctor privately without her partner “knowing everything.” Taken together, these findings suggest that mobile phones may provide a degree of safety and autonomy for young Indigenous people who use drugs.

### Addressing Mobile Connectivity Challenges

Continuity of phone connection was often not consistently available to Cedar participants via a mobile phone and plan [2,35]. Our survey did not differentiate between having a phone versus having a phone with a phone number and airtime credit. However, when asked if they had a phone, many participants produced a handset, but explained that it was not connected except through Wi-Fi. Through informal discussions that emerged while administering this survey, we learned that mobile phone handsets are fairly accessible to participants and they reported getting phones from various sources, including second hand, as gifts, and through trades. However, substantial barriers to maintaining cellular connectivity exist, including high costs and existing debts with providers, that prevent participants from having a phone plan [33,35]. Of note, the costs of cellular services are notably higher in Canada than in other international markets, especially when the most basic packages are compared [73]. Gonzales [4] offers the concept of “technology maintenance” to highlight the time, energy, and money required to maintain phone connectivity even after a phone handset is acquired and uses the term “dependable instability” to refer to the frequent, short-term disconnections among low-income people in the United States. Many Cedar participants may experience such periods as a result of jail, housing transitions, relationship breakdowns, phone loss, lack of phone credit, or missed payments. Participants identified the resultant loss of touch with support and service networks as significant barriers to access. In her study of 37 low-income clients attending two free health clinics in the US Midwest, Gonzales et al [2] found that short-term phonelessness contributed to lost employment, lost welfare benefits, and strains on social support networks, which she describes as critical for health. The authors argued that frequently changing mobile phone numbers may disrupt access to health services, resources, and social support [2]. Homeless adults in Philadelphia also characterized phones as being important tools to fulfill responsibilities to work, housing, and social support—all powerful social determinants of health [35,74].

In the absence of a phone number where they can be reached consistently over time, many participants use social media and messaging apps (eg, Facebook) that can be accessed via Wi-Fi on a smart phone that does not have a cellular plan. High use (90%) of Facebook and other Web-based communication tools have been observed among other marginalized groups, including street-involved youth in Denver [34], British Columbia [33], and Seattle [75]. When a phone is lost, stolen, or disconnected, these points of digital connection are not severed in the same way as when the phone number is cancelled. However, at present, it is not common practice for health care providers to connect using these alternative digital technologies, perhaps because of institutional policies limiting the use of social media [2]. Reaching clients via messaging apps other than SMS may be a useful way to stay connected despite phone disruptions. Previous research has suggested that the success of mHealth programs that rely on text messages may be a result of capitalizing on technology that is both familiar and part of regular habits and routines, rather than creating something new (such as a new mobile app) that requires behavior change [76]. Given that many participants had access to a mobile phone without a cellular plan, future programs may be interested in using Web-based messaging programs that have better continuity in the event of phone loss or missed payments.

### Recommendations for Future Mobile Health Programs

Taken together, the findings discussed so far emphasize the importance of addressing ongoing phone access and connectivity within future mHealth programs involving young Indigenous people who use drugs. In addition, participants’ perspectives support growing evidence that two-way, open-ended supportive mHealth interventions are more effective than those that are more narrowly focused (eg, reminders only, single health condition) [77-79]. First, participants voiced a broad view of their health, which captures physical, emotional, mental, and family wellness. This is affirmed by studies that articulate a diversity of health priorities among Indigenous people living with/affected by HIV, beyond those directly related to the virus [80,81]. Others have described a variety of strategies Indigenous people living with HIV use to stay healthy that include, but are not limited to, taking antiretroviral therapy (ART) medications [57]. Narrowly focusing on HIV/AIDS prevention and treatment outcomes, such as substance use recovery or ART adherence, may limit the potential for mHealth programs to address a person’s own health priorities and goals. Further, frameworks developed by Indigenous bodies to guide mental wellness and substance use services in British Columbia and Canada call for programs that build on the principle of wholistic wellness [48,82]. Second, highly targeted approaches, such as text message reminders to take a specific medication such as ART or methadone may disclose the participants’ HIV status or drug use if intercepted. Open-ended text messages, such as the “how are you?” approach taken in the original WelTel Kenya trial minimizes the possibility of disclosure by allowing participants to direct the conversation according to their comfort level [13]. In the context of ongoing stigma and criminalization of drug use and HIV in Canada and elsewhere, avoiding unwanted disclosure is essential [83,84]. However, the participants reported few concerns in this regard. It is possible that lack of concern stemmed from the fact that participants already constantly navigate privacy and disclosure related to drug use and HIV status and have established strategies they would apply in the mHealth context.

### Limitations

This study has several potential limitations. The cross-sectional design limits the identification of trends or causal associations. Our sample may not be representative of all young Indigenous people who have used illicit drugs in British Columbia; however, efforts were made to ensure that a diversity of characteristics were represented, including gender, city of residence, and injection and noninjection drug use. Further, Indigenous peoples are diverse, and as this survey involved a particularly vulnerable group of young Indigenous people who have used drugs, findings cannot be generalized to young Indigenous people in general. Finally, our survey did not capture participants’ use of mobile handsets that were not connected via a cellular plan. However, this has emerged as an area for future research.

### Conclusions

While interest in using mobile phones for health is high among young Indigenous people who have used drugs in British Columbia, low rates of phone ownership present a barrier to engagement in mHealth. Future mHealth programs will need to take this into account, either by providing mobile phone handsets and cellular plans, by supporting texting through Web-based platforms currently utilized by their client base or innovating to reduce periods of phonelessness and/or loss of connectivity. Mobile phones were viewed as a way to support connections and relationships that are seen as critical to health and well-being among the young Indigenous people in this study. In addition, participants articulated a wholistic view of health that included physical, mental, emotional, and family well-being. As a result, open-ended mHealth initiatives for young Indigenous people who have used drugs that strengthen relationships with care providers and other social supports, enable individuals to set their own priorities for health and well-being, and take a culturally safe approach are recommended.

## Abbreviations

ART: antiretroviral therapy
mHealth: mobile health
